# Monoclonal gammopathy progressing to systemic amyloidosis with cardiac involvement. A case report

**DOI:** 10.47487/apcyccv.v6i2.477

**Published:** 2025-06-27

**Authors:** Germán Valenzuela-Rodríguez, Ofelia Araoz Tarco, Iván Fernández Vertiz

**Affiliations:** 1 Clínica Delgado, Lima, Peru. Clínica Delgado Lima Peru; 2 Unidad de Revisiones Sistemáticas y Metaanálisis (URSIGET), Vicerrectorado de Investigación, Universidad San Ignacio de Loyola, Lima, Peru. Universidad San Ignacio de Loyola Unidad de Revisiones Sistemáticas y Metaanálisis (URSIGET) Vicerrectorado de Investigación Universidad San Ignacio de Loyola Lima Peru; 3 Instituto Nacional Cardiovascular INCOR, Lima, Peru. Instituto Nacional Cardiovascular INCOR Lima Peru; 4 Hospital Nacional Edgardo Rebagliati Martins, Lima, Peru. Hospital Nacional Edgardo Rebagliati Martins Lima Peru

**Keywords:** Light Chain Amyloidosis, Cardiac, Monoclonal Gammopathy of Undetermined Significance, Amiloidosis de Cadenas Ligeras de las Inmunoglobulinas, Corazón, Gammapatía Monoclonal de Relevancia Indeterminada

## Abstract

We report a case of systemic light chain amyloidosis with cardiac involvement, preceded by a monoclonal IgG lambda gammopathy. The clinical diagnosis was based on signs of heart failure, elevated cardiac biomarkers, and characteristic imaging findings. The diagnosis was confirmed by increased levels of free light chains in blood and urine, as well as the presence of amyloid deposits in periumbilical fat and multiple segments of the gastrointestinal tract. Treatment with daratumumab, bortezomib, and dexamethasone was initiated, followed by autologous hematopoietic stem cell transplantation 22 months after diagnosis, resulting in a favourable clinical outcome.

## RESUMEN

Reportamos el caso de una amiloidosis sistémica de cadenas ligeras con compromiso cardíaco, precedida de una gammapatía monoclonal por IgG lambda. El diagnóstico clínico se realizó por signos de insuficiencia cardíaca, elevación de biomarcadores y hallazgos de imágenes, orientándose con la elevación de cadenas ligeras en sangre y orina, y confirmándose con la presencia de depósitos de amiloide en la biopsia de grasa periumbilical y en diferentes segmentos del tracto digestivo. Se inició tratamiento con daratumumab más bortezomib más dexametasona, recibiendo un transplante de células madre hematopoyéticas de tipo autólogo a los 22 meses del diagnóstico, con una evolución clínica favorable.

## Introduction

Primary light chain amyloidosis is a plasma cell neoplasm characterised by the presence of monoclonal plasma cells and amyloid light chain deposits in various organs, including the kidneys (46%), heart (30%), liver (9%), gastrointestinal tract (7%), peripheral nerves (5%), and soft tissues (3%). Cardiac involvement is considered an adverse prognostic factor for survival, making early detection critical ^1^^,^^2^.

Diagnosis requires histological demonstration of amyloid protein, although cardiac biopsy is rarely necessary. In 90% of cases, amyloid deposits are detected in periumbilical adipose tissue aspirates or bone marrow biopsies. However, if organ biopsy is needed, renal biopsy offers a diagnostic sensitivity greater than 95%, while endomyocardial biopsy approaches nearly 100% ^3^. This condition may arise from other entities, such as monoclonal IgG lambda gammopathy, although this is exceedingly rare ^4^^,^^5^.

## Case report

A 57-year-old woman with a history of diffuse abdominal pain lasting more than five years, hyperinsulinaemia, venous insufficiency, and a six-year history of monoclonal IgG lambda gammopathy without criteria for multiple myeloma, presented to the emergency department with a two-day history of dyspnoea, fever up to 39 °C, and cough. She experienced rapid clinical deterioration in the emergency area and was transferred to the intensive care unit with an oxygen saturation of 80% despite high-flow oxygen therapy, prompting initiation of mechanical ventilation. She also developed sustained hypotension with a blood pressure of 70/50 mmHg, requiring vasopressor support. On clinical examination, she had mild lower limb oedema, normally coloured skin and mucous membranes, crackles at the bases of both lungs, and rhythmic heart sounds of low intensity without murmurs

**Figure 1:**
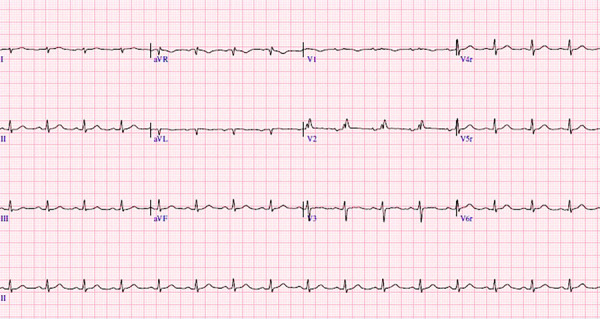
Twelve-lead electrocardiogram showing low-voltage QRS complexes, incomplete right bundle branch block, and overall reduced voltage.

**Figure 2:**
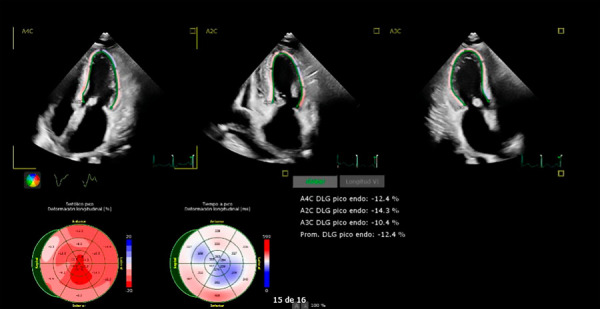
Echocardiogram demonstrating reduced global longitudinal strain (-12.4) at the time of diagnosis

A chest computed tomography scan revealed pneumonia in the left lower lobe, for which meropenem was initiated. The patient showed a slow but favourable clinical course over a 14-day period. Moreover, the electrocardiogram demonstrated low-voltage QRS complexes (Figure 1), and there was a marked elevation of N-terminal pro-B-type natriuretic peptid (NT-proBNP) during hospitalisation, from 5486 pg/mL to 6151 pg/mL (normal range: 0-300 pg/mL), along with an increase in troponin T from 0.51 ng/mL to 1.03 ng/mL (normal range: 0-0.05 ng/mL). Transthoracic echocardiography showed a left ventricular ejection fraction of 70%, left atrial area of 19 cm² (normal: < 20 cm²), interventricular septum thickness of 11 mm (normal: < 10 mm), posterior wall thickness of 12 mm (normal: < 10 mm), and an indexed left ventricular mass of 72 g/m² (normal: 44-95 g/m²). The myocardium appeared hyperrefringent, with a mild posterolateral pericardial effusion of 4 mm, and the global longitudinal strain was reduced to -12.4% (Figure 2).

**Figure 3:**
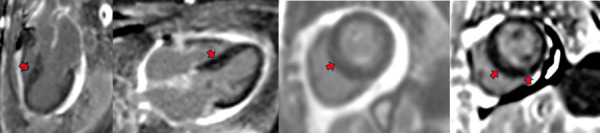
Cardiac magnetic resonance imaging in T1 inversion recovery (IR) sequences, showing late gadolinium enhancement in three-chamber, four-chamber, and short-axis views. Intramyocardial (focal) fibrosis is observed, predominantly in the interventricular septum and inferior wall (red arrows), with a distribution that is atypical for cardiac amyloidosis.

Based on the electrocardiographic, laboratory, and imaging findings, cardiac amyloidosis was suspected. Cardiac magnetic resonance imaging was subsequently performed, revealing the following: preserved left ventricular systolic function (58.6%); normal global and segmental wall motion; no evidence of intramyocardial oedema; late gadolinium enhancement predominantly affecting the interventricular septum and inferior wall; preserved right ventricular systolic function (52.9%); and a mild pericardial effusion (lateral: 4.7 mm, inferior: 7 mm) without haemodynamic compromise (Figure 3).

**Figure 4:**
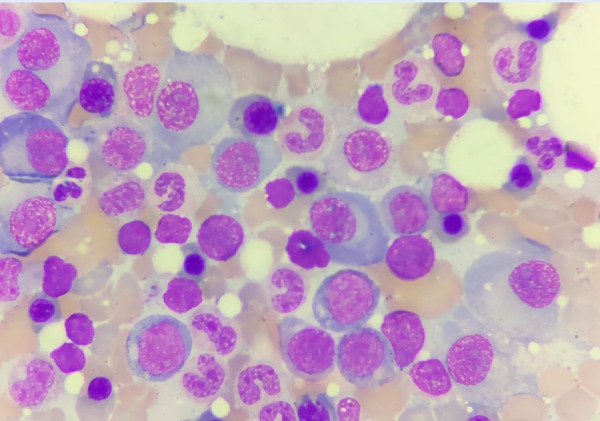
Area of aberrant plasma cells identified by bone marrow flow cytometry, expressing clonality with the following phenotype: cIgG Lambda ++ / cIgG Kappa - / CD38+ / CD56 +++ / CD20 - / CD117 ++.

Additional tests were performed, including antinuclear antibodies (ANA), antineutrophil cytoplasmic antibodies (ANCA), and an antiphospholipid panel, all of which were negative. Bence Jones proteins in 24-hour urine were negative. Serum kappa light chains were 7.78 mg/L (normal range: 3.3-19.4), while serum lambda light chains were 1645.2 mg/L (normal range: 5.71-26.3). Urinary kappa light chains were 81.06 mg/L (normal range: 0.012-32.71), and urinary lambda light chains were 121.4 mg (normal range: 0.01-4.99). Lactate dehydrogenase was 207 U/L (normal range: 135-214). Immunoglobulin A and M levels were within normal limits. Bone marrow analysis revealed an area of aberrant plasma cells identified by flow cytometry (Figure 4).

Periumbilical adipose tissue biopsy was positive for Congo red staining, confirming the diagnosis. Upper endoscopy and colonoscopy were performed due to epigastric burning and loose stools. Congo red staining was positive in biopsy samples from the gastric lamina propria, duodenal lamina propria, ascending colon, transverse colon, descending colon, and rectum.

The patient showed favourable clinical progression and was discharged home with furosemide 20 mg daily, pantoprazole 40 mg on an empty stomach, sucralfate 5 mL 30 minutes before bedtime, and *Bacillus clausii* spores, one ampoule orally every 8 hours for 7 days. The final diagnosis was systemic amyloidosis, clinical stage IV (Mayo staging system) ^6^, with cardiac and gastrointestinal involvement. Treatment was initiated with daratumumab, bortezomib, and dexamethasone. During follow-up, the patient developed atrial flutter, for which apixaban 2.5 mg every 12 hours was started.

**Figure 5:**
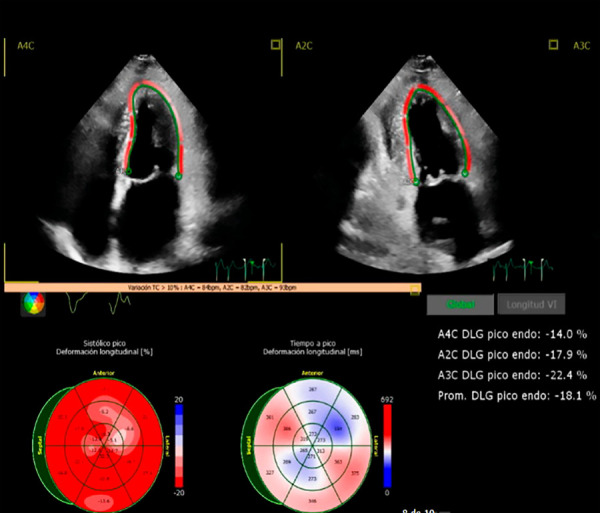
Echocardiogram showing improvement in global longitudinal strain two years after diagnosis.

At 22 months after diagnosis, the patient underwent autologous hematopoietic stem cell transplantation, with a favourable clinical course. From a cardiovascular perspective, she showed marked clinical improvement, including a reduction in NT-proBNP levels and improvement in strain rate on echocardiography (Figure 5).

## Discussion

This case involves a patient with non-IgM monoclonal gammopathy who progressed to systemic amyloidosis with severe cardiac involvement. In terms of disease progression, the annual risk of developing other conditions such as non-Hodgkin lymphoma, chronic lymphocytic leukaemia, or multiple myeloma is approximately 1%, with progression to light chain (AL) amyloidosis and plasmacytomas occurring less frequently ^4^^,^^5^.

Cardiac amyloidosis is classified into several types, each with distinct cardiac and extracardiac manifestations. The three primary forms are AL amyloidosis and transthyretin amyloidosis, which includes the wild-type form (wtATTR) and hereditary variants (hATTR) ^6^^,^^7^.

AL amyloidosis is rare, with an incidence of 1 per 100,000 in the United States, and is associated with plasma cell dyscrasias. It typically affects patients from the fourth decade of life and presents with symptoms such as elevated jugular venous pressure, hepatic congestion, and right ventricular failure. Advanced stages are associated with low-output syndromes, including decreased pulse pressure and delayed capillary refill ^6^^,^^7^. Electrocardiographic findings often show low voltage in the limb leads and a pseudo-infarct pattern in precordial leads, despite echocardiographic evidence of increased left ventricular wall thickness ^6^^-^^12^.

In AL amyloidosis, misfolded light chain proteins are deposited in the extracellular space of the cardiac muscle, leading to impaired myocardial function. The primary goal of treatment is to halt the production of these abnormal proteins. However, factors such as advanced age, increased risk of bleeding, thromboembolic events, and multiorgan involvement contribute to an overall poor prognosis ^7^^,^^13^. Additionally, atrial arrhythmias are more prevalent in cardiac amyloidosis than in the general population, reaching up to 44% in wild-type transthyretin amyloidosis, particularly in advanced stages of the disease ^14^.

### Therapeutic options include:

- The CyBorD chemotherapy regimen, which consists of cyclophosphamide, bortezomib, and dexamethasone ^15^^,^^16^.

- Autologous hematopoietic stem cell transplantation following high-dose melphalan. Eligibility for this treatment generally requires the patient to be under 70 years of age, without significant organ dysfunction, with good performance status (Eastern Cooperative Oncology Group [ECOG] < 2), New York Heart Association (NYHA) functional class < 3, and biomarker levels below established thresholds ^15^.

- Daratumumab, a human IgG monoclonal antibody targeting CD38, recently approved by the US Food and Drug Administration (FDA) in combination with the CyBorD chemotherapy regimen, based on data from the ANDROMEDA clinical trial ^15^.

- Alternative regimens for patients who are not eligible for transplantation, cannot tolerate bortezomib, or lack access to daratumumab include oral melphalan and dexamethasone, which have shown partial haematologic responses in at least 60% of patients ^15^^,^^16^.

For patients with refractory amyloidosis or relapse following initial treatment, options include daratumumab monotherapy; proteasome inhibitor-based regimens such as ixazomib with dexamethasone; immunomodulatory agents including lenalidomide, thalidomide, and pomalidomide; and salvage therapy with bendamustine. Monoclonal antibodies such as CAEL-101 and birtamimab are currently under investigation ^15^^,^^17^.

In conclusion, this case represents an atypical progression to systemic amyloidosis with cardiac involvement in a woman with a prior monoclonal gammopathy. Clinical symptoms and cardiovascular diagnostic findings guided the early investigation for amyloid deposits. Cardiac involvement, which is associated with increased morbidity and mortality, requires chemotherapy and, when available, hematopoietic stem cell transplantation.
